# Operative Fixation of Pediatric Forearm Fractures: Does the Fracture Location Matter?

**DOI:** 10.1155/2021/9973449

**Published:** 2021-10-16

**Authors:** Ahmed Elabd, Ramy Khalifa, Zainab Alam, Ehab S. Saleh, Ahmed M Thabet, Amr Abdelgawad

**Affiliations:** ^1^Department of Orthopaedic Surgery, Medstar Washington Hospital Center, Washington, DC, USA; ^2^Department of Orthopaedic Surgery and Rehabilitation, TTUHSC-El Paso, Paul L. Foster SOM, Elpaso, TX, USA; ^3^Department of Orthopaedic Surgery, Oakland University William Beaumont School of Medicine, Rochester, MI, USA; ^4^Department of Orthopaedic Surgery, Maimonides Medical Center, Brooklyn, NY11204, USA

## Abstract

**Background:**

Flexible intramedullary nails (FNs) are successfully used to treat pediatric forearm fractures, especially midshaft fractures. Distal forearm fractures have been described as “difficult to manage” with FN insertion. The purpose of this study was to report the clinical and radiographic outcomes of using flexible nails in pediatric forearm fractures and the impact of fracture location on the outcome of the procedure.

**Methods:**

This is a retrospective review of pediatric patients who presented with forearm fractures that were surgically treated with flexible nails between 2009 and 2018. Patient demographics, fracture location, and classification were reported. Intraoperative and postoperative complications were reported. The primary outcomes were fracture radiographic union, intraop and postop complications, and the need for additional surgical procedures.

**Results:**

Fifty-nine patients were included, with a mean age of 11 years. All fractures healed with patients regaining full range of motion. The authors were able to use flexible nails successfully in 48/59 (81%) patients. In eleven cases (19%), FN fixation was not able to provide adequate fixation to maintain reduction. The method of fixation was changed from FN insertion to another method in nine cases. In two cases, FN fixation was augmented with another fixation method. Fractures within 3 inches of the distal articular surface were at a higher risk of intraoperative change/augmentation of the fixation method (29%) compared with fractures that occurred more than 3 inches from the distal articular surface (11%).

**Conclusion:**

The majority of pediatric forearm fractures can be treated successfully with flexible nails. Surgeons involved in treating these fractures should pay attention to distal third fractures. Stabilizing the distally located fractures using FN fixation can be challenging. Surgeons should be prepared to use an alternative fixation method when needed.

## 1. Introduction

Forearm fractures are common fractures among the pediatric population. They account for approximately 18% of all pediatric fractures [1]. Traditionally, these fractures have been treated with closed reduction and casting. Recently, there is an increasing trend toward operative treatment to avoid the complications associated with nonoperative treatment including malunion, loss of reduction, and limited forearm rotation [2]. The operative indications include open fractures, failure to obtain or maintain adequate closed reduction, compartment syndrome, floating elbow, and displaced fractures in older children near skeletal maturity [3].

Flexible nail (FN) has been successfully used as a fixation method for pediatric forearm fractures [4]. Schmittenbecher reported the effect on fracture location on the outcome of using FN in pediatric forearm fractures. The authors reported that the distally located pediatric forearm fractures were more prone to loss of reduction compared with the diaphyseal and proximal pediatric forearm fractures. This may be attributed to the distal radial fragment usually being too short to be sufficiently held by the nail. The larger medullary canal will not be adequately filled with the FN to maintain the reduction [5]. Other authors have proposed that FN fixation is not a good option for distal forearm fractures [6, 7]. Other alternative fixation methods include plating, percutaneous Kirschner wire (K-wire) osteosynthesis, and external fixation.

The purpose of this study was to report the clinical and radiographic outcomes of using flexible nails in pediatric forearm fractures and the effect of fracture location on the use of flexible nails in distal third fractures.

## 2. Materials and Methods

The study was an institutional review board (IRB) approved, retrospective chart, and radiograph review of all pediatric forearm fractures. The study included all operatively treated at forearm fractures at level I pediatric trauma center between 2009 and 2018. The study group included all patients aged between 7 and 18 years treated with surgical fixation of forearm fractures. Exclusion criteria were patients older than 18 years or those who were primarily treated with other fixation methods. The study variables included patient demographics, time to surgery, operating surgeon, and operative reports. Radiographs were reviewed for fracture characteristics: fractured bone (radius, ulna, or both); fracture location; and fracture type (open or closed). Distal third fractures were defined as fractures that occur within 3 inches from the distal articular surface. Intraoperative variables included method of fixation and type of reduction (open or closed). The majority of forearm fractures at our institution were treated nonoperatively by closed reduction and castings. Nearly all fractures in children under the age of eight years are treated by closed reduction and casting. Operative fixation was only indicated for fractures that we were unable to obtain and/or maintain acceptable reduction by closed means, most displaced fractures in patients older than 11 years in female or 13 years in male (much less remodeling potential), open fractures, and floating elbow [3].

The primary outcomes were fracture radiographic union, intraop and postop complications, and the need for additional surgical procedures. Acceptable reduction was dependent on the patient age. For patients older than 8 years, our limit for accepted reduction was 15° angulation for distal shaft fractures, 10° for more proximal fractures, and 50% apposition [8]. The need for open reduction to restore fracture alignment was reported. All operations were performed by two surgeons who were fellowship-trained in both orthopedic trauma and pediatric orthopedics.

### 2.1. Surgical Technique

All surgical interventions were done under general anesthesia. The entry point for the distal radius was the dorso lateral aspect of the radius just proximal to the distal radius physis (which was identified by fluoroscopy. Deep dissection was in-between the first and second dorsal compartment. A drill hole was performed proximal to the distal radius, and the hole was enlarged using an awl. A flexible nail (2 or 2.5 mm depending on the patient age/size) was introduced in a retrograde fashion. A trial of closed reduction was done by gentle traction and gentle manipulation. If the reduction and nail passage were not achieved using few trials, open reduction of the fracture was done using a small incision centered over the fracture. Two bone clamps were used to reduce the fracture, and then the nail was passed from one side to the other. If reduction could not be maintained using FN fixation (more than 50% overlap), the surgeon either decided to add another FN to maintain better reduction, add crossing K-wire fixation, or abandon the FN and use plate fixation. This decision to augment or change the fixation method was considered “additional surgical procedure.” After radius fixation, the ulnar fracture was assessed. If it was reduced and stable, it was not surgically fixed. If, on the contrary, the ulnar fracture was still displaced and/or unstable, ulnar fixation using antegrade nailing was done. Small incision was done distal to the ulnar physis (physeal sparing), and then FN was passed from the proximal to distal end of the fracture.

### 2.2. Statistical Analysis

Quantitative variables were described using means and standard deviations. Categorical variables were described using frequencies and proportions. Student's *t*-test and the chi-squared test were used to assess differences in changes of fixation methods. Linear regression models with the Poisson family and link log were used to assess the unadjusted association between change in fixation and selected cofactors. These were reported as prevalence ratios (PRs) and 95% confidence intervals (CIs). A *P* value < 0.05 was considered statistically significant. All analyses were conducted using Stata 15 (StataCorp LLC, College Station, Texas, USA). The statistical work was reviewed by the Statistical Department of the School of Medicine to ensure correct methodology.

## 3. Results

This study included 59 patients (42 male and 17 female patients) with 59 fractures. The mean patient age at the time of surgery was 11 years (range: 7–16 years). The fracture location was the distal third in 24 fractures (41%), and 35 fractures were in the middle and proximal (59%). According to the Gustilo–Anderson classification, six (10%) of the fractures were open fractures: type I (5/59) and type II (1/59). The time from injury to surgical intervention was an average of 3 days (range: 0–18 days).

Forty-eight (81%) of the 59 procedures were successfully completed using FN insertion (requiring either closed or open reduction). The surgeons' satisfaction with the reduction quality and stability was recorded in the operative reports. Among the 48 cases, 22 (46%) entailed closed reduction, and 26 (54%) required open reduction of at least one bone.

**Figure 1:**
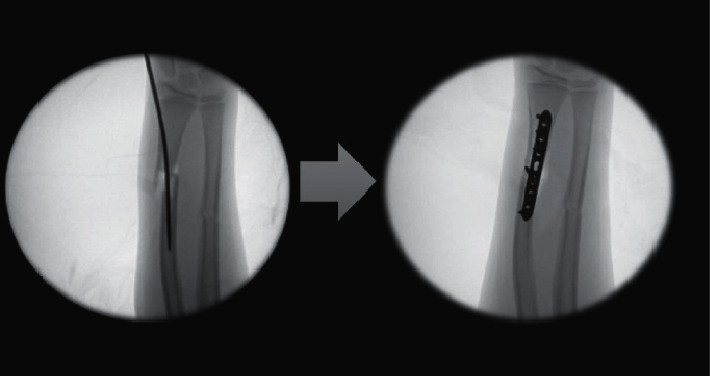
Intraoperative fluoroscopic images showing residual fracture displacement after passage of the FN. The fixation method was changed to plate fixation, and anatomical reduction was obtained.

**Figure 2:**
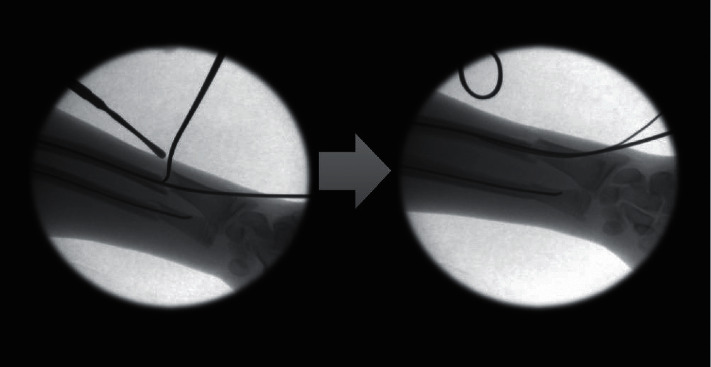
Intraoperative fluoroscopic images showing residual fracture displacement after passage of the FN. Augmentation of fixation was pursued with another FN.

**Figure 3:**
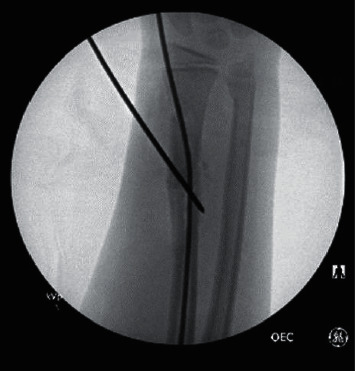
Intraoperative fluoroscopic image showing fixation augmentation with a K-wire.

Eleven (19%) of the 59 cases who required either a change of the method of fixation or augmentation of the FN fixation of the radius were identified. Among nine of the 11 cases, the method of fixation was changed intraoperatively to plate fixation in eight cases (Figure 1) and K-wire fixation in one. In the remaining two cases, the surgeon augmented the FN fixation because the reduction continued to be displaced with single FN. The augmentation was accomplished with another small nail in one case (2 nails across the fracture) (Figure 2) and with crossing K-wires in the other (Figure 3).

Seven out of these 11 cases (64%) were located at the distal third of the shaft. This represented 29% of distal forearm fractures (7 out of 24). Four of the 11 cases (34%) that required further intervention were located more than 3 inches from the distal articular surface (mid/proximal shaft fractures). These 4 cases represented only 11% of the mid/proximal shaft fractures (4 out of 35). All cases which required change/augmentation were related to the radius bone. A summary of the results is presented in Table 1.

The findings of statistical analysis of variables in relation to changing the method of fixation from FN insertion to other methods were reported as PR, 95% CI, and *P* values. Age and fracture classification had no statistically significant relation to intraoperative failure. Fractures within 3 inches of the distal articular surface were at a higher risk of intraoperative change/augmentation of the fixation method (29%) compared with fractures that occurred more than 3 inches from the distal articular surface (11%) (PR = 1.28; CI: 1.06–1.56; *P*=0.012).

## 4. Discussion

Forearm fractures in children are a common reason for emergency department visits [1]. Most pediatric forearm fractures are treated nonoperatively with closed reduction and immobilization with a cast or splint [9, 10]. Operative fixation is indicated for certain fractures [3, 8]. Fixation options include FN fixation, small-fragment plate fixation, and K-wire fixation [11]. FN insertion is becoming more popular because of the less invasive nature of the procedure, shorter operative time, and excellent functional outcomes [1, 4].

The current study has an important finding that the distally located fracture is more prone to reduction difficulty or even failure when fixation with FN was elected as the fixation method compared to other radius fractures in the pediatric population. In the current study, all fractures healed with few complications. All patients returned back to preoperative activity levels. No additional surgical procedures were needed. The majority of fractures (81%) were successfully stabilized intraoperatively with FN insertion as preoperatively planned. However, the surgeons needed to change or augment the method of fixation in approximately one-fifth of the patients, 19%. The change to different fixation methods or augmentation represents the inability of the single FN to maintain the reduction. The change of the fixation method occurred more in distal third fractures (29%). This important finding was echoed by other authors [5, 7, 12, 13]. Cai et al. [12] described the distal radial metaphyseal fractures as “difficult to manage” because of the geometry of that area. In the classic intramedullary nailing practice, the entry point is close to the fracture plane, and the elasticity of the nail usually pushes the proximal fragment toward the contralateral side, thereby potentially leading to angulation and malalignment. Additionally, Kim et al. [13] studied FN insertion as a method of fixation of distal metadiaphyseal junction forearm fractures in adolescents. They determined that the minimal distance between the fracture line and the distal articular surface should be > 3.5 cm for FN fixation to be considered. If a fracture site was located ≤3.5 cm from the physis, it was considered unsuitable for FN insertion.

The current study showed that the use of FN insertion to treat pediatric forearm fractures is not a panacea, especially for fractures of the distal part of the shaft. Inability to use the flexible nails to maintain reduction in nearly one-third of the distally located fractures (29%) was observed. By reviewing the operative reports and intraoperative fluoroscopic images, we tried to identify the possible causes of failure. In some cases, after insertion of the FN, the fracture was still notably displaced (Figures 1 and 2) with minimal apposition. The remaining displacement was because of the distal location of the fracture with a wide medulla, the progression of the fracture line from the insertion point to the original fracture, or the marked fracture comminution—either traumatic or iatrogenic—from the repeated trial of reduction and nail insertion.

Moreover, some authors have described distal forearm fractures as not optimum for FN [5, 7]. In 2005, Schmittenbecher [5] evaluated the treatment procedures, problems, complications, and final results of pediatric forearm fractures going back to 1976. Successful treatment of these fractures depends on the selection of the correct treatment modality. It is not only the decision between nonoperative and operative treatment but also the choice of the implant. Schmittenbecher indicated that the FN is the first choice for midshaft and proximal fractures; however, K-wires are often preferred in distal diaphyseal or diaphyseal-metaphyseal fractures. It is acceptable if the surgeon considers plate fixation as the primary treatment in females who have started menstrual periods and in males older than 13 years. Slongo [7] discussed the complications and failures associated with the FN technique, addressing system-related and fracture-related problems separately. The main causes of FN failure when used to treat forearm fractures were small nail diameter and lack of correct tensioning of the two nails against each other. However, the greatest problems occurred when the FN was inappropriately used for radial fractures in the distal third and metaphyseal regions. Slongo considered those fractures not ideal indications for FN insertion. Recently, Du and Han [14] introduced a new operative approach to treat irreducible distal radius fracture in the diaphyseal-metaphyseal junction with satisfactory outcomes. They used antegrade FN technique from the proximal radius with an entry point located 2.4 cm distal to the proximal articular surface of the radius at the dorsolateral aspect (Thompson approach). The authors noted that the classic retrograde insertion of FN is not possible for distal fractures [15].

**Figure 4:**
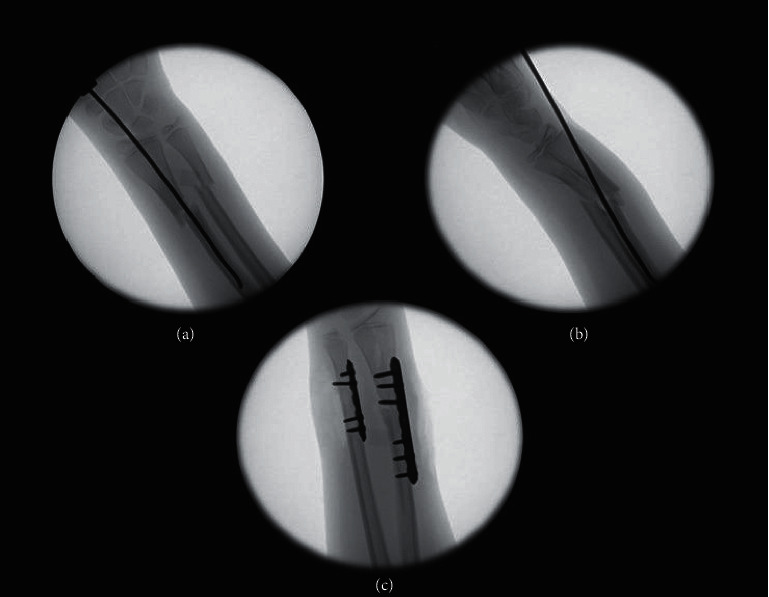
(a, b) Intraoperative fluoroscopic images showing two views of flexion deformity and translation at the fracture site induced by ulnar entry of the FN. (c) Intraoperative fluoroscopic image showing the change of the fixation method to plate osteosynthesis.

It is worth noting that the failure of FN fixation to maintain reduction of the distal forearm fracture was described in the *Nancy University Manual*, in which this form of treatment was first introduced to orthopedic surgeons [15]. The authors of the manual noted that when the FN is introduced from its usual dorsoradial entry point in a distal fracture, it displaces the fracture, very similar to our findings. For this reason, they advised against using this entry point and recommended an ulnar entry point for these distal fractures. Using the modified entry point, in our experience, did not completely solve the problem of fracture displacement in all cases because doing so can cause flexion of the distal fragment in exchange for correcting the coronal alignment problems associated with using the radial entry point (Figure 4).

All operations were performed by two surgeons who were fellowship-trained in both orthopedic trauma and pediatric orthopedics. Failure to maintain satisfactory reduction is therefore not likely to be attributable to the lack of surgical training or knowledge. Our current approach to the distal third of the radius and ulnar fractures is a single trial of fixation using an FN. If, after the passage of the nail, the fracture is still significantly displaced, plate fixation should be considered.

The main limitation of our study is the retrospective nature of the study and small number in each group. However, even with the relatively small number of each group, the authors were able to find a statistically and clinically significant impact of fractures' location on using FN in pediatric forearm fractures.

## 5. Conclusions

The majority of pediatric forearm fractures can be treated successfully with flexible nails. Surgeons involved in treating these fractures should pay attention to distal third fractures. Stabilizing the distally located fractures using FN fixation can be challenging. The study results show that FN fixation of distally located forearm fractures (compared with proximal and midshaft fractures) has a higher chance of inability to maintain fracture reduction requiring utilization of another fixation method. Surgeons planning to use FN in distal forearm fractures should be prepared to use an alternative fixation method.

## Figures and Tables

**Table 1:** Summary of the results.

*N*	Successful	Further intervention	Total	Success (%)	Failure (%)
Distal 1/3	17	7	24	71	29
Proximal 2/3	31	4	35	89	11
Total	48	11	59	81	19

## Data Availability

The data used to support the findings of this study are included within the article.
